# Notes and Comments

**Published:** 1894-09

**Authors:** 


### Notes and Comments.[1]

Development of Character.—Can one overcome temperament? is a question often forced upon the thoughtful mind. If the question is asked with enough earnestness to lead one to try the experiment on himself, he will thereafter have more to say about self-training than home training. It is admitted that inherited traits can be modified, if not overcome. Temper, disagreeable voice or gesture, an ungraceful walk, a tendency to untruthfulness—all traits that weaken or mar character—are being constantly effaced by those who recognize inherited burdens. If this were not so, we would ask, with a recent writer in the *Outlook*, what would we mean by development of character?

A Word concerning Discussion and Criticism.—The definition of the verb to discuss is to shake apart, so as to examine thoroughly; in fact, it is taken from a Latin word which means to shake. When a body of men and women are brought together for mutual benefit, as is the object of our society meetings, it would be well if the literal meaning of the word were always the real significance given it in practice. Unfortunately in some of our meetings many of the speakers seem to have no such object in view. At times the proceedings read as though the sole purpose of the society was to further the art of mutual admiration; then, again, one would think it was the report of a verbal pugilistic performance. Happily, however, a higher appreciation of society meetings is being developed, and it is desirable as far as possible that personalities should be eliminated; but when one feels justified in terming an essay "elementary" (see page 580), it should be known that it is the essay, not the essayist, that is referred to. And, further, it is well to remember the words of Lowell, where he says, "When Mr. Mathew Arnold charges Shakespeare with exaggeration, it shall not set me in a passion, but put me upon honest inquiry, rather, to find out whether the fault is in the poet or the critic."

---

[1] The assistant editor solicits contributions for this department,—new methods, new remedies and formulas, or any short practical note which may prove of value to the practitioner or student. Address 212 South Fifteenth Street, Philadelphia.

The Assimilation of Minerals.—A recent writer in the *Items of Interest* has truly said that we have little power to assimilate minerals taken as mere separate chemicals. It is principally by their being assimilated first into vegetable life that they are capable of becoming an integral part of our animal economy; they are made still more easily digested by us if they come from the vegetable through the higher organization of the lower animals. The flinty rock becomes the silica of the teeth by becoming first the silica of the wheat. Iron is hard to chew, and almost as hard to digest as a powder, but presented as beefsteak it easily enters every tissue of the body.

